# Inter-rater reliability of needle EMG pattern classification among physicians

**DOI:** 10.3389/fneur.2026.1982862

**Published:** 2026-10-15

**Authors:** Ekaterina Seliverstova, Artem Pyatkov, Mariya Kovalchuk, Evgenia Druzhinina, Mikhail Sinkin

**Affiliations:** 1 I.V. Davydovsky City Clinical Hospital of the Moscow Department of Health, Moscow, Russia; 2 Burnasyan Federal Medical Biophysical Center of Federal Medical Biological Agency, Moscow, Russia; 3 I.M. Sechenov First Moscow State Medical University (Sechenov University), Moscow, Russia; 4 Neuro-muscular Disorders Clinic, Moscow, Russia; 5 Pirogov Russian National Research Medical University, Moscow, Russia; 6 N.V. Sklifosovsky Research Institute for Emergency Medicine, Moscow, Russia

**Keywords:** needle electromyography, EMG, inter rater reliability, myogenic, neurogenic, clinical experience, GRRAS, motor unit potential

## Abstract

**Objective:**

To evaluate the inter-rater reliability of needle electromyographic (EMG) assessment among 19 physicians with varying clinical experience and specialties using a consensus standard as reference.

**Methods:**

The study followed the GRRAS guidelines. First, four EMG experts agreed on classifying thirty EMG recordings as normal, myogenic, or neurogenic and referred to as a consensus standard (CS). Second, those anonymised recordings were independently classified by 19 non-expert physicians. Thereafter, Cohen’s kappa (κ) was calculated for each physician against the CS. Fleiss’ κ was used for multi-rater agreement. Category-specific (one-vs-rest) κ, sensitivity and specificity were computed. Subgroup analyses by experience and specialty were performed. Post-hoc power analysis was used for the specialty comparison.

**Results:**

The mean κ versus the consensus standard across all physicians was 0.688 (median 0.747, SD 0.172; range 0.287–0.900). Overall multi-rater agreement (Fleiss’ κ) was 0.581. Category-specific κ values were: neurogenic 0.723, normal 0.368, myogenic 0.334. Sensitivity/specificity: neurogenic 94.6%/87.1%, myogenic 69.7%/96.1%, normal 78.2%/88.9%. Agreement with the CS increased with experience: mean κ rose from 0.365 (group 1, ≤1 year) to 0.798 (group 4, >10 years). No significant difference was found between neurologists and clinical physiologists (Mann–Whitney U, *p* = 0.174); post-hoc power was low (≈45%), thus non-significance should not be interpreted as equivalence. EMG experts showed almost perfect agreement (*κ* = 0.85–0.95).

**Conclusion:**

In this exploratory study, physicians demonstrated moderate to substantial agreement with a CS standard in EMG classification. Neurogenic changes are identified most reliably, whereas differentiation between normal and myogenic changes remains challenging. Greater experience was associated with better agreement in our sample, but this finding requires confirmation in larger studies. No statistically significant difference was found between neurologists and clinical physiologists, although the analysis was underpowered. Individual competency varied widely within specialties, suggesting that objective, competency-based qualification may be more relevant than specialty alone.

## 1 Introduction

Needle electromyography (EMG) is crucial in the evaluation of neuromuscular disorders, enabling differentiation between neurogenic and myogenic changes and identification of normal findings. The diagnostic utility of needle EMG heavily depends on the interpreter’s experience and the consistency of pattern classification. While several studies have examined inter-rater reliability in lumbar radiculopathy (1–3), limited evidence exists regarding overall pattern classification (normal/myogenic/neurogenic) among physicians with different backgrounds, especially without access to clinical data.

Across countries, physicians from various medical backgrounds are permitted to perform needle EMG: technicians/clinical neurophysiologists/neurologists with special education in neurophysiology. Not only the level of experience, but also basic clinical education may influence the interpretation of needle EMG.

The main aim was to assess the inter-rater reliability of needle EMG pattern classification among 19 physicians with varying experience and specialties, compared with a consensus standard (CS) established by four EMG experts. Secondary objectives included: (1) evaluating individual physician agreement with the CS; (2) quantifying overall multi-rater agreement; (3) computing category-specific reliability, sensitivity, and specificity; and (4) exploring the influence of clinical experience and specialty on agreement. Given the exploratory design, no formal hypothesis testing for subgroup differences was pre-specified.

## 2 Methods

The study followed the Guidelines for Reporting Reliability and Agreement Studies (GRRAS) (4) (Figure 1).

**Figure 1:**
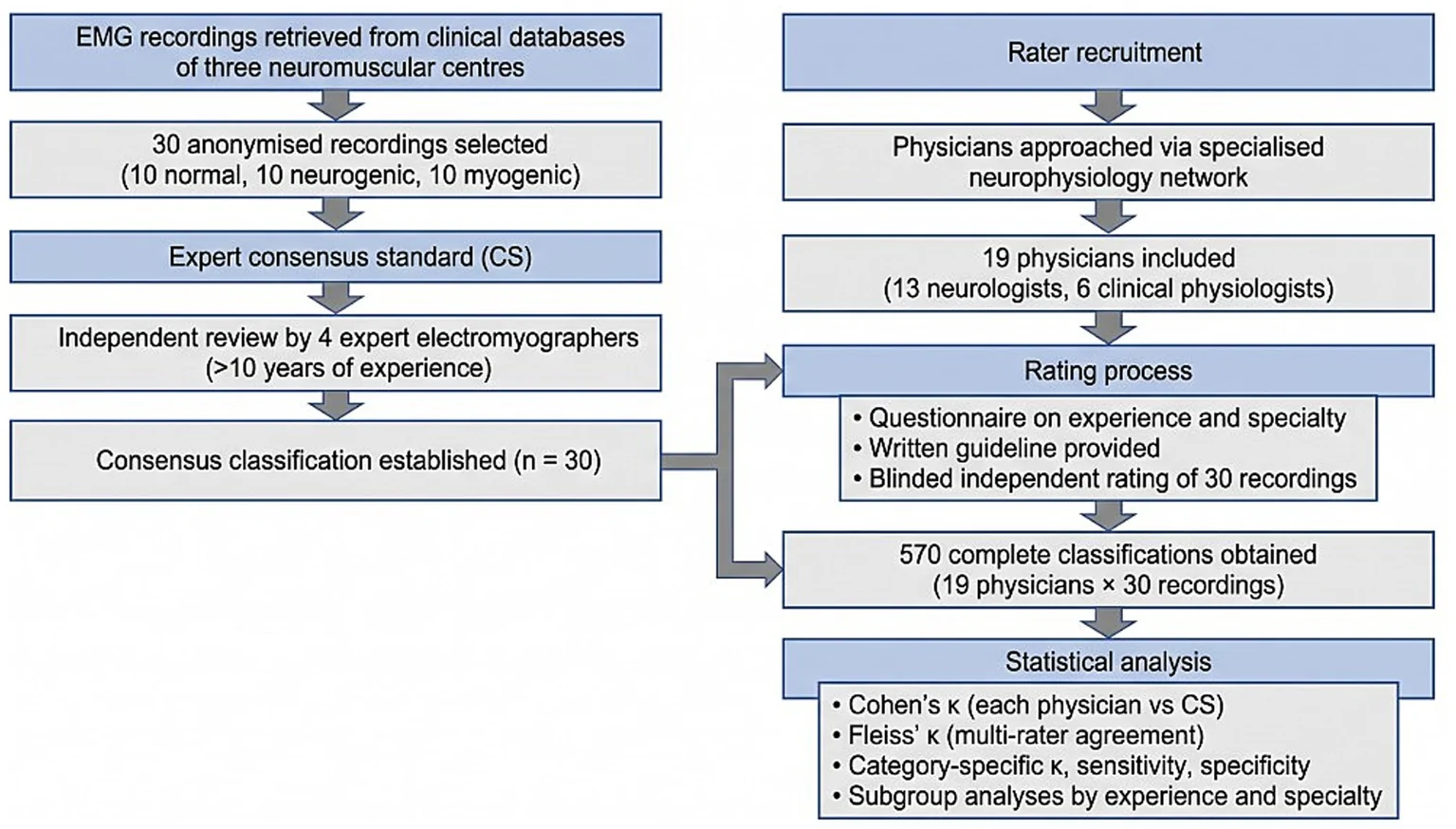
Flow diagram of the study process.

### 2.1 Data collection

Anonymised EMG datasets of thirty patients, previously classified by the expert consensus standard (CS) as normal (*n* = 10), neurogenic (*n* = 10) or myogenic (*n* = 10), were obtained from the clinical databases of three neuromuscular departments. Each dataset (one “recording” = one patient-level examination) had been acquired by one of three EMG experts (ED, ES or AP) on an EMG machine (“LITEBOX,” Neurosoft®) with a disposable concentric needle electrode (“B50600,” l = 50 mm, d = 0.45 mm, Neurosoft®) following standard methodology (5). The protocol muscles were deltoid, biceps brachii, extensor digitorum communis, first dorsal interosseous, vastus lateralis, tibialis anterior and gastrocnemius; not every muscle was available in every patient. A case was included if the sampled muscles permitted a pattern-level judgement and a minimum of six motor unit potentials (MUPs) had been extracted per sampled muscle. Six MUPs was set as the operational minimum because in a severe neurogenic process very few motor units remain available for sampling; requiring the conventional ~20 MUPs for formal quantitative analysis would have excluded precisely those neurogenic recordings that the study needed to represent. The unit of analysis for all subsequent ratings was the patient-level pattern (normal/myogenic/neurogenic), not a single muscle.

The consensus standard (CS) was established independently of the original acquisition session. Four expert electromyographers (ED, ES, AP and MK)—neurologists specialising in neuromuscular disorders, with international neurophysiology training, membership of neurophysiology/neuromuscular societies and >10 years of EMG experience—independently re-classified each of the 30 anonymised datasets as normal, myogenic or neurogenic. Disagreements were then resolved in a consensus meeting. CS is therefore an electrophysiological consensus on the MUP pattern of the dataset, not the treating physician’s nosological diagnosis. A case was labelled neurogenic or myogenic when the overall pattern across sampled muscles showed that change; isolated borderline findings in a single muscle were not sufficient. Classification used standard qualitative MUP features (duration, amplitude, phases and the recruitment impression available on the stored traces), consistent with IFCN quantitative EMG standards (6). Individual expert agreement with the final CS (reported in Results) refers to each expert’s independent pre-consensus rating and is not an external validation of the same panel against itself.

The recordings were uploaded to cloud storage without pre-placed MUP marker overlays (“free of MUP bounding”), without the interference curve, without clinical or demographic data, and without separately scored insertional activity or force-graded recruitment. The assigned task was to place MUP markers, assess duration and amplitude of the motor-unit potentials, and classify each recording as normal, myogenic or neurogenic on the basis of those MUP features. Demographic and clinical data cannot be retrieved: the files were exported from the EMG software in anonymised form.

### 2.2 Non-experts rating process

19 non-experts were approached via specialized neurophysiology chat to participate in retrospective EMG analysis. They were first asked to complete a questionnaire on their EMG experience and medical background (neurology or clinical physiology), followed by a guideline on assessing the recordings (Supplementary File S1).

They were deprived of clinical or demographic data. Non-experts were asked to classify each recording as “normal,” “myogenic,” or “neurogenic”. All 19 physicians were blinded to one another’s classifications and to the CS. One month was allowed for completion. The 19 participating physicians were not involved in constructing the CS. After all 19 responses had been collected, each of the four EMG experts’ independent pre-consensus ratings was used only to describe expert-level agreement with the final CS and mutual agreement among experts.

### 2.3 Power considerations

No *a priori* power calculation was performed because this was an exploratory study. However, using the method of Sim and Wright (7) and an expected Cohen’s κ of 0.6 for two raters with three categories, we estimate that at least 50 samples would be required for stable κ estimation with 19 physicians. Thus, our sample of 30 recordings provides limited precision, especially for subgroup analyses. This limitation is addressed in the Discussion. For the comparison between neurologists and clinical physiologists, a post-hoc power analysis was conducted.

### 2.4 Comparison with GRRAS guidelines

Our study adhered to most GRRAS items. Key elements added in this revision include: detailed rater characteristics, description of the rating process and blinding, ethics approval, data availability statement, and power considerations. The main remaining limitation is the small sample size, which is explicitly acknowledged.

### 2.5 Statistical analysis

Cohen’s kappa (κ) was calculated for each physician versus the CS. For agreement among the four EMG experts and for multi-rater agreement among the 19 physicians, Fleiss’ κ was used. Category-specific (one-versus-rest) Cohen’s κ values were computed on the pooled 570 classifications (19 physicians × 30 recordings) against the CS; this is the sole category-specific κ reported in this manuscript (Table 1). Pairwise Cohen’s κ among the 19 physicians (not Pearson correlation) was used to visualise rater similarity (Figure 2), with hierarchical clustering applied to the κ matrix. Sensitivity and specificity were derived from the same aggregated 3 × 3 contingency table. Because the 570 classifications are clustered within recordings and within raters, they are not statistically independent; sensitivity and specificity are therefore reported as descriptive pooled estimates rather than as inferential parameters.

**Table 1:** Category-specific κ (physicians vs. CS).

Category	κ (95% CI)*
Neurogenic	0.723 (0.68–0.77)
Normal	0.368 (0.31–0.43)
Myogenic	0.334 (0.28–0.39)

*Bootstrap 95% confidence interval (1,000 iterations).

**Figure 2:**
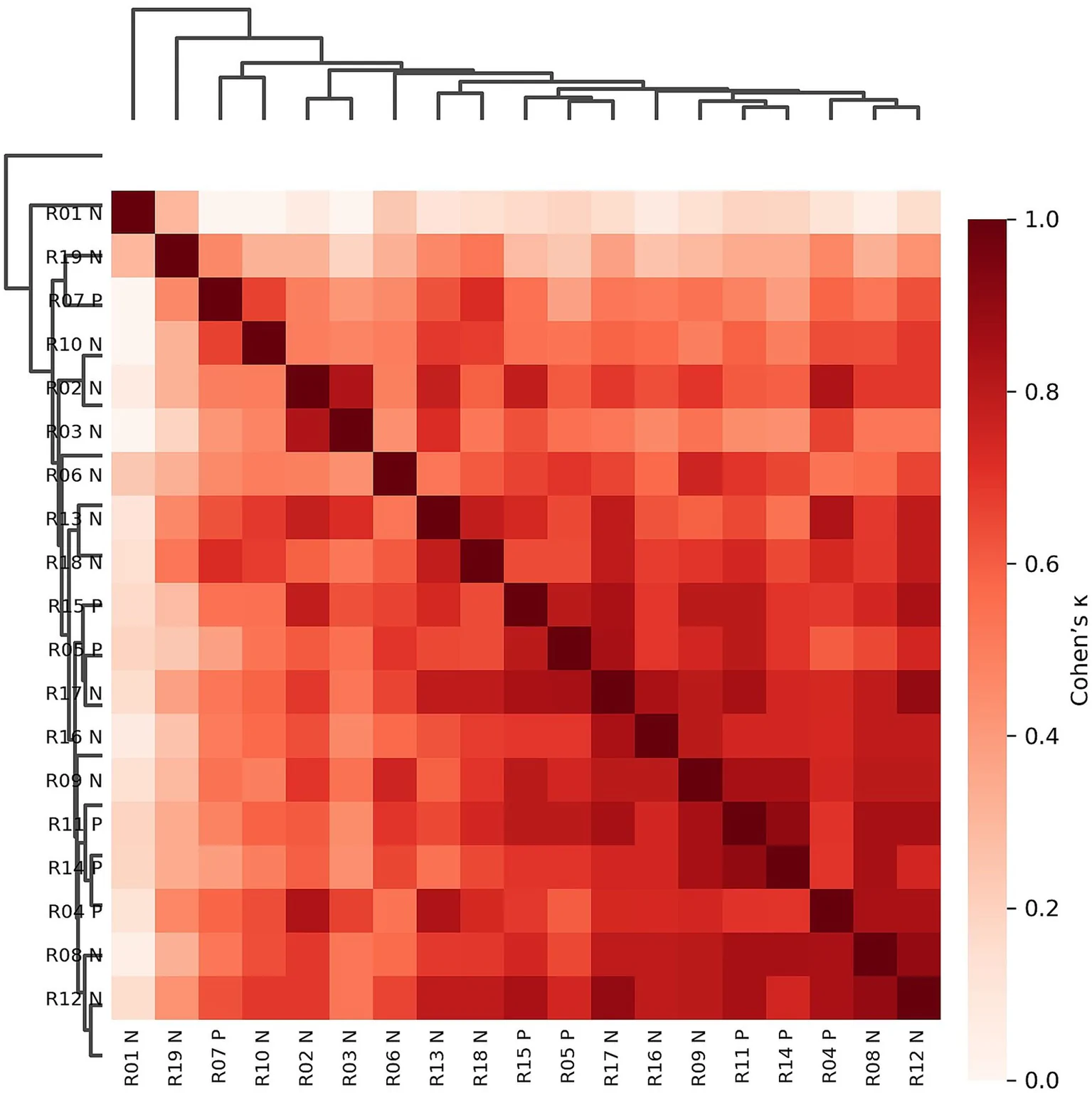
Pairwise Cohen’s κ matrix of classifications from 19 physicians (R01–R19; *N* = neurologist, P = clinical physiologist). Colour intensity reflects the magnitude of pairwise agreement (average-linkage clustering on distance = 1 − κ). Mean off-diagonal *κ* = 0.58.

Subgroup analyses were performed for experience groups and specialties. The Mann–Whitney *U* test was used to compare κ distributions between neurologists and clinical physiologists. A post-hoc power analysis for the specialty comparison was performed using GPower (version 3.1) with an effect size Cohen’s d = 0.5 (observed from the data), *α* = 0.05, and sample sizes n1 = 13, n2 = 6.

All analyses were performed using the *SPSS* 26 application package and Keptune data analysis AI for Excel, CSVs & Databases.

Bootstrap 95% confidence intervals (1,000 iterations) were calculated for selected κ values.

### 2.6 Ethics statement

All EMG recordings were obtained from patients who had previously given written informed consent for the scientific use of their anonymised data. This is a retrospective analysis of existing anonymised neurophysiological recordings; no additional procedures were performed for the study.

## 3 Results

### 3.1 Experts

The four experts’ independent pre-consensus ratings showed consistently high agreement with the final CS (κ ≥ 0.85) and substantial mutual agreement (Fleiss’ κ = 0.85).

### 3.2 Non-experts

Most physicians (52.6%) had 1–3 years of needle EMG experience; 21.1% had >10 years. The majority were neurologists (13/19, 68.4%); the remainder were clinical physiologists without a neurological background (6/19, 31.6%). For subgroup analyses, physicians were grouped into four experience categories based on the numeric code in the original dataset (Figure 3).

- Group 1: ≤1 year (*n* = 2)
- Group 2: 2–3 years (*n* = 10)
- Group 3: 4–9 years (*n* = 3)
- Group 4: >10 years (*n* = 4)

**Figure 3:**
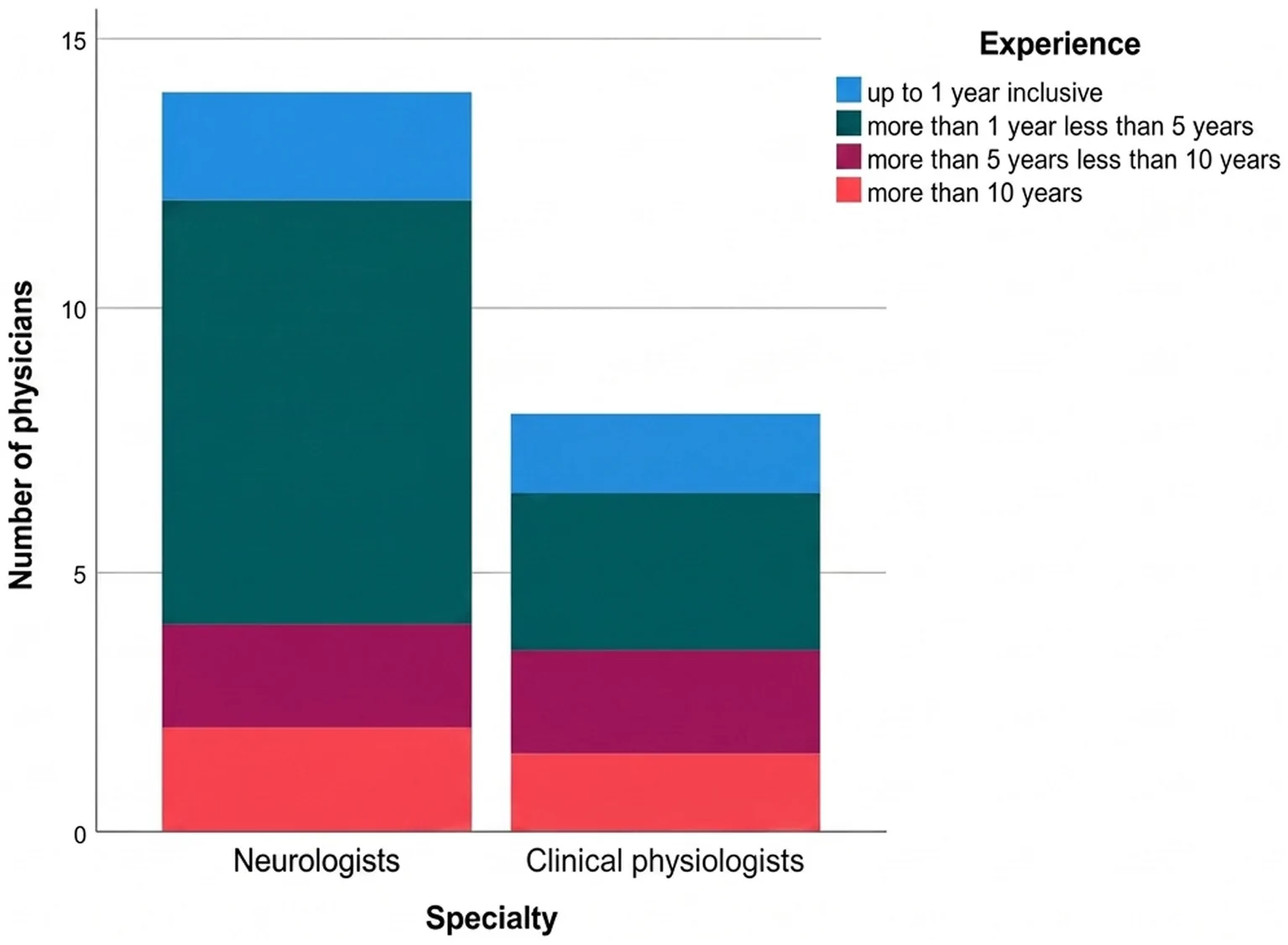
Distribution of physicians by experience level and specialty: bar chart showing counts for each experience group, with colour coding for neurologists vs. clinical physiologists.

Due to the small size of Groups 1 and 3, all subgroup comparisons are exploratory and should be interpreted with caution.

### 3.3 Agreement with the consensus standard

Cohen’s κ for each of the 19 physicians versus CS is summarised in Table 2.

**Table 2:** Summary of Cohen’s κ (physicians vs. CS).

Metric	Value
Number of physicians	19
Median κ	0.747
Min—max κ	0.287–0.900

Pairwise Cohen’s κ among the 19 physicians showed predominantly moderate agreement (mean off-diagonal κ = 0.58), indicating a fair-to-moderate level of inter-physician concordance in diagnostic choices (Figure 2). Hierarchical clustering of the κ matrix identified several coherent groups of physicians with highly similar classification patterns, while a small number of outliers (notably R01) demonstrated markedly lower concordance with the rest of the cohort.

### 3.4 Overall multi-rater agreement (Fleiss’ κ)

For all 19 non-experts and 30 samples, Fleiss’ κ was 0.581 (moderate agreement). The observed overall agreement P̄ was 0.729, while chance-expected agreement Pe was 0.354. The marginal proportions of category assignments were: normal – 0.332, myogenic – 0.232, neurogenic – 0.437.

### 3.5 Category-specific agreement (one-vs-rest κ)

Table 1 reports category-specific (one-versus-rest) Cohen’s κ computed on the pooled 570 classifications (19 physicians × 30 recordings) against the CS. The highest agreement was achieved for the neurogenic MUPs (*κ* = 0.723), whereas normal and myogenic patterns showed only fair agreement (*κ* = 0.368 and 0.334, respectively).

Bootstrap 95% CI for the mean individual Cohen’s κ (all physicians vs. CS) was 0.608–0.764. Category-specific one-versus-rest κ values are those in Table 1 only (neurogenic 0.723; normal 0.368; myogenic 0.334). No alternative category-specific κ set is reported.

### 3.6 Diagnostic accuracy (sensitivity and specificity)

To describe diagnostic performance versus the CS, all 570 classifications (19 physicians × 30 recordings) were aggregated into a single 3 × 3 contingency table. Sensitivity and specificity were calculated for each diagnostic category using a one-versus-rest approach and are presented as descriptive pooled estimates (see Methods). Relative to the CS, physician judgements had the highest sensitivity for the neurogenic pattern (94.6%) and the highest specificity for the myogenic pattern (96.1%) (Table 3).

**Table 3:** Sensitivity and specificity of physician classifications versus the consensus standard (CS).

Category	Sensitivity (%)	Specificity (%)
Myogenic	69.7	96.1
Neurogenic	94.6	87.1
Normal	78.2	88.9

Sensitivity: proportion of CS-positive cases labelled as that category. Specificity: proportion of CS-negative cases not labelled as that category. Values are descriptive pooled estimates (570 clustered classifications).

### 3.7 Pooled confusion matrices by specialty group (vs. CS)

To examine systematic classification differences between neurologists (*n* = 13) and clinical physiologists (*n* = 6), we aggregated all individual predictions within each group across the 30 samples. The total number of decisions was 390 for the neurologist group and 180 for the other group.

The proportion of true myogenic samples misclassified as neurogenic was substantially higher among neurologists (14.5%) than among clinical physiologists (3.7%). In contrast, the reverse error (true neurogenic called myogenic) remained low in both groups (2.8% for neurologists, 1.5% for clinical physiologists). Neurogenic recognition was excellent in both groups (92.3% for neurologists, 97.0% for clinical physiologists). Myogenic recognition was considerably better in the other physicians group (77.8% vs. 61.5% for neurologists). Normal samples were identified with comparable accuracy (≈75%) by both groups (Figure 4).

**Figure 4:**
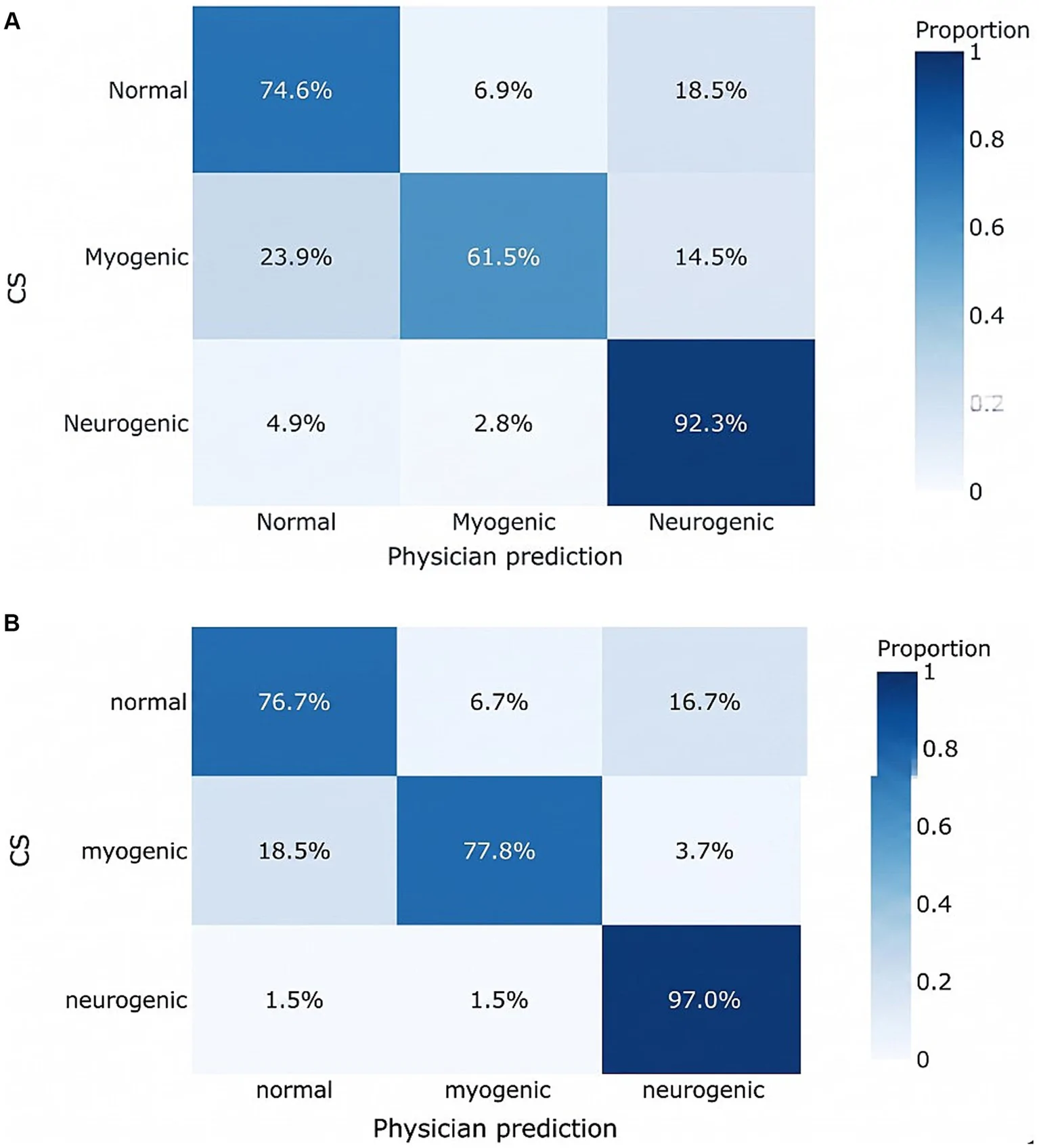
Confusion matrices for neurologists [**(A)**, *n* = 13, 390 decisions] and for clinical physiologists [**(B)**, *n* = 6, 180 decisions] when compared with the CS. Rows represent true CS categories; columns represent physician predictions. Numbers inside cells are percentages of the true class (row percentages). Colour intensity is proportional to the cell percentage.

### 3.8 Subgroup analysis by clinical experience

Table 4 summarises performance by experience group (exploratory analysis). Mean Cohen’s κ versus the CS and Fleiss’ κ increased with experience: from 0.365 and 0.297 (group 1) to 0.798 and 0.755 (group 4). Group 2 (2–3 years) unexpectedly outperformed group 3 (4–9 years). This counter-intuitive finding is most plausibly due to the small size of group 3 (*n* = 3) and possible heterogeneity within that group rather than a genuine mid-career decline. Groups 1 and 3 are too small for stable inference; Table 4 is therefore descriptive only.

**Table 4:** Exploratory performance by experience group (descriptive only; Groups 1 and 3 are small).

Group	Experience	n	Mean κ vs. CS	Fleiss’ κ (95% CI)
1	≤1 year	2	0.365 (0.110)	0.297 (0.15–0.44)
2	2–3 years	10	**0.752** (0.139)	**0.695** (0.62–0.77)
3	4–9 years	3	0.543 (0.052)	0.537 (0.44–0.63)
4	>10 years	4	**0.798** (0.042)	**0.755** (0.68–0.83)

Groups 1 and 3 are small; CIs for Fleiss’ κ should be interpreted cautiously.

The subgroup analysis suggests a positive association between years of needle EMG practice and diagnostic agreement with the CS. Physicians with the longest experience (Group 4, >10 years) achieved the highest mean Cohen’s κ (0.798), indicating substantial to almost perfect agreement, whereas those with the least experience (Group 1, ≤1 year) showed only fair agreement (mean *κ* = 0.365). The intra-group agreement measured by Fleiss’ κ followed a similar trend: Group 4 highest (0.755), Group 2 high (0.695), Group 3 moderate (0.537), Group 1 low (0.297) (Figure 5). Thus, in this sample, more experienced physicians both matched the CS more closely and agreed more with each other. These comparisons remain exploratory.

**Figure 5:**
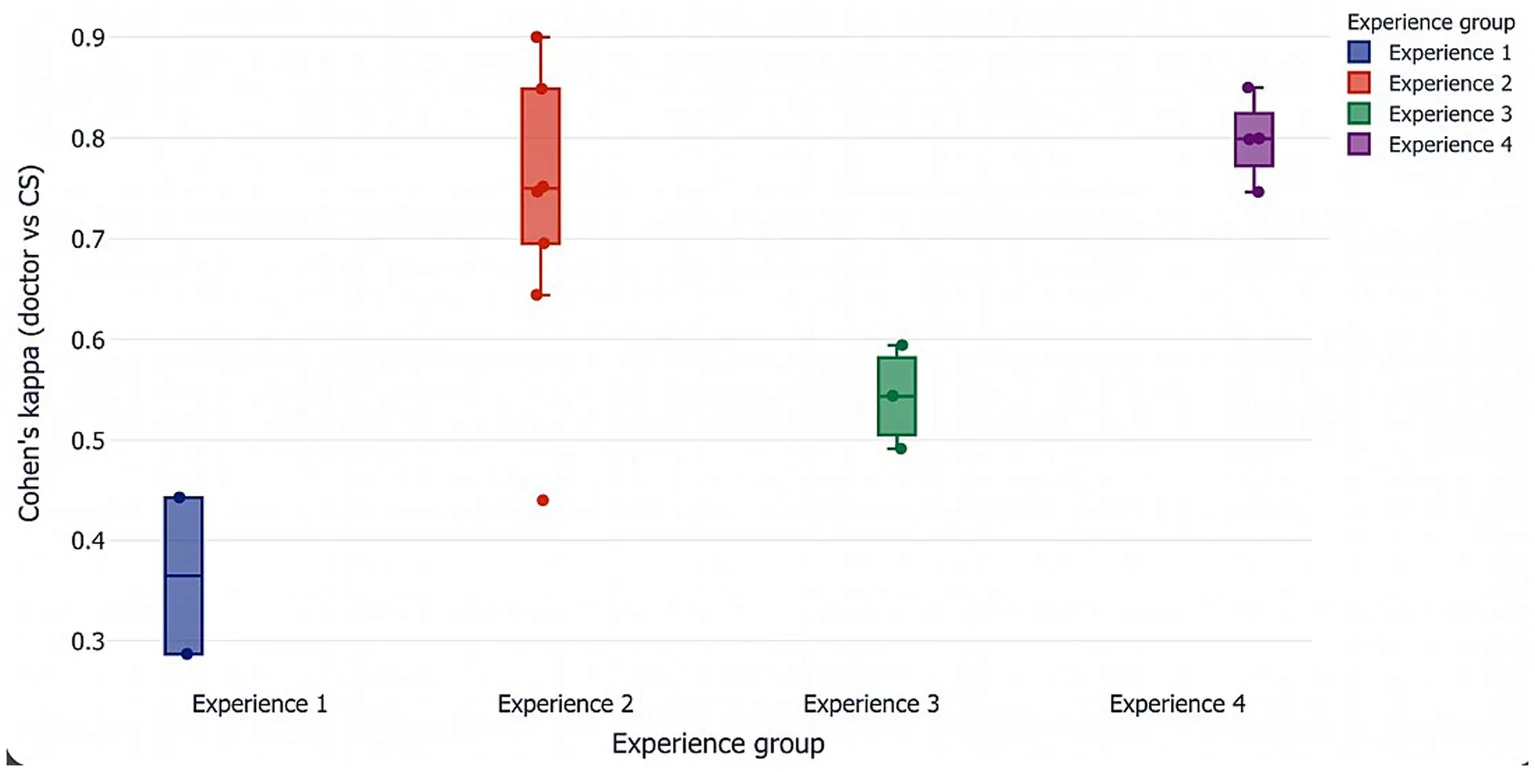
Distribution of Cohen’s Kappa vs. CS by experience group.

A limitation is the small sample size in Groups 1 and 3, which reduces statistical power. Confidence intervals for Fleiss’ κ in these groups are approximate; a study with at least 10–15 physicians per experience level would be needed to confirm the learning curve.

### 3.9 Comparison by specialty

The comparison of individual Cohen’s κ values (physicians vs. CS) between neurologists (*n* = 13) and clinical physiologists (*n* = 6) revealed no statistically significant difference (Mann–Whitney U = 23.0, *p* = 0.174). Although the median κ was numerically higher in the clinical physiologists group (0.799 vs. 0.746), the overlap in interquartile ranges (neurologists: 0.544–0.752; clinical physiologists: 0.721–0.875) and the relatively small sample size in the clinical physiologists group likely contributed to the lack of significance (Figure 6). A post-hoc power analysis revealed that with the observed effect size (Cohen’s d ≈ 0.5) and group sizes (13 vs. 6), the achieved power was approximately 45%. Thus, the non-significant *p*-value should not be interpreted as evidence of equivalence. A larger study with at least 20 physicians per specialty would be needed to detect a similar difference with 80% power.

**Figure 6:**
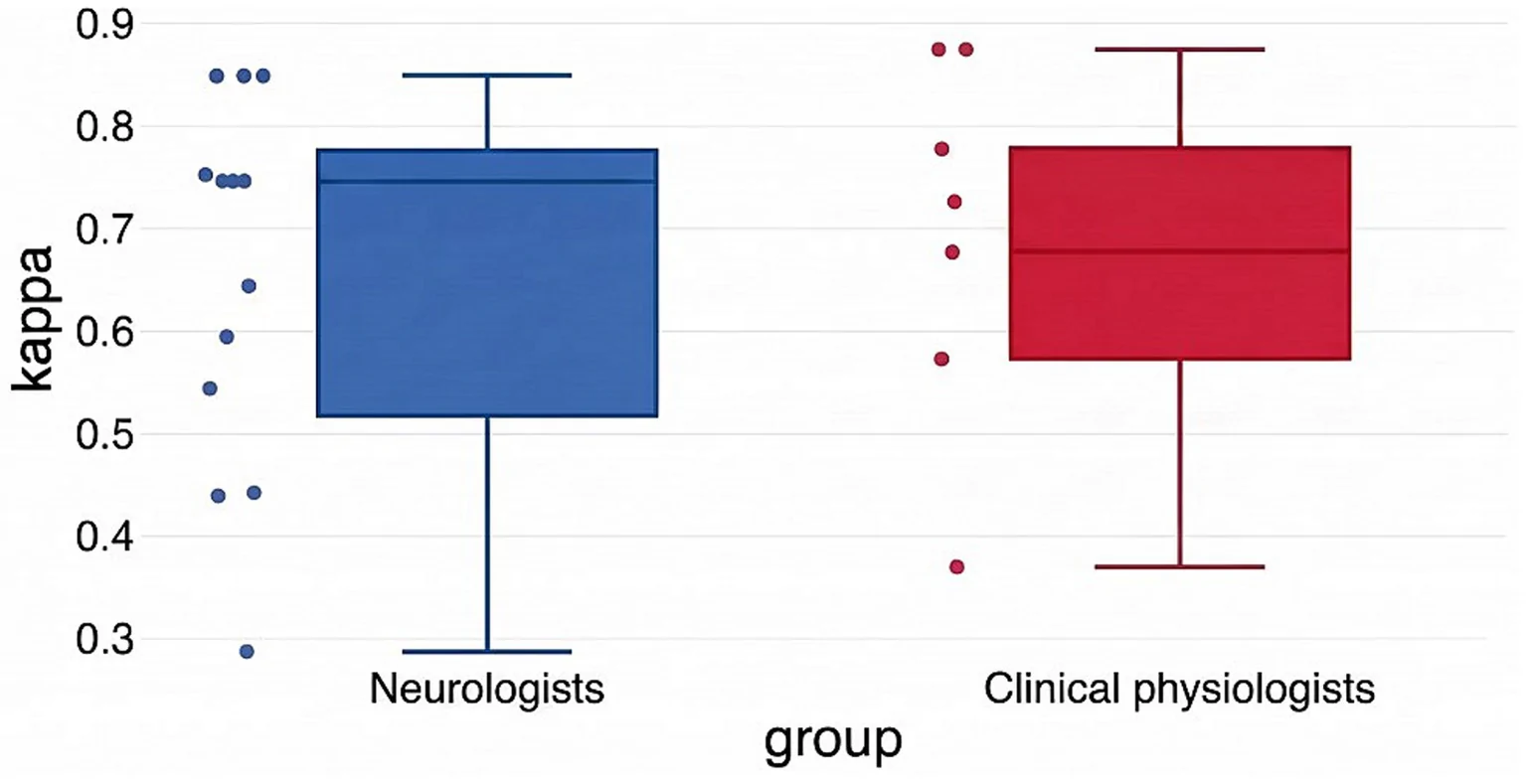
Boxplot of Cohen’s κ values for neurologists vs. clinical physiologists: Medians, interquartile ranges, and individual points shown. No significant difference, but power is limited.

## 4 Discussion

In this study we examined how consistently physicians of different specialties and levels of experience classify needle EMG recordings as normal, myogenic or neurogenic in the absence of clinical information. Compared with a consensus standard established by four experts, overall agreement among the 19 physicians was moderate to substantial. First, the three EMG patterns proved to be of unequal diagnostic difficulty: neurogenic changes were recognised with high reliability, whereas differentiation between normal and myogenic patterns remained challenging. Second, individual agreement with the consensus standard varied considerably and was associated with clinical experience but not with specialty affiliation.

Our results are broadly consistent with earlier investigations of inter-rater reliability in needle EMG. Kendall and Werner (1) reported only fair agreement between blinded examiners and an expert interpreter in the setting of lumbosacral radiculopathy, with certified and more experienced raters performing substantially better than uncertified residents do. Chouteau et al. (2), who restricted their sample to certified experts assessing spontaneous activity, found substantial agreement. The present study is closer in design to that of Kendall and Werner (1) in that the raters were deliberately heterogeneous in experience and specialty. At the same time, the classification task was more demanding, requiring discrimination among three mutually exclusive patterns rather than a binary judgement of radiculopathy.

Taken together, these findings suggest that agreement is relatively high when the diagnostic question is coarse (neurogenic versus non-neurogenic) but declines when finer distinctions are required. Chronic neurogenic reorganization produces high-amplitude, long-duration and often polyphasic MUPs together with reduced recruitment features that are visually conspicuous and repeatedly emphasized during training. Myogenic changes, by contrast, are frequently subtle. Short, low-amplitude polyphasic potentials may lie close to the normal range, so that classification depends heavily on quantitative thresholds rather than on the presence or absence of an obvious abnormality. Without numerical reference values, raters rely on a holistic visual impression that is least reliable at the boundary between normal and mild myopathy. This interpretation is supported by the International Federation of Clinical Neurophysiology (IFCN) standards, which underline the importance of comparison with normative data and acknowledge the limited availability of well-defined reference values in routine practice (6). In the present series, specificity for the myogenic pattern was high, yet sensitivity remained comparatively low, indicating that physicians rarely over-called myopathy but frequently under-called it. Occasional misclassification of truly myogenic recordings as neurogenic may reflect erroneous placement of motor-unit potential markers that artificially prolongs measured duration, leading to an impression of neurogenic reorganisation.

Raters were not provided with the interference curve or with force-graded recruitment. The traces were stored without MUP marker overlays. Each physician independently placed markers on the motor-unit potentials, assessed MUP duration and amplitude, and classified the recording as normal, myogenic or neurogenic on the basis of those MUP features. The study therefore evaluates inter-rater reliability of marker placement and of MUP-based pattern assignment, not of a complete bedside EMG. Insertional and spontaneous activity and the clinical question that guides muscle selection were likewise unavailable. The comparatively low sensitivity for the myogenic class is consistent with this restricted task, because myopathic change is often recognised in practice from the interference pattern at a given force rather than from a small sample of isolated MUPs alone. The conventional target of approximately 20 MUPs per muscle for formal quantitative MUAP analysis was not required. Six MUPs was the operational minimum, because in a severe neurogenic process very few motor units remain and a larger sample cannot be obtained. This choice preserves the neurogenic end of the spectrum but may still limit recognition of mild myopathic change, for which a larger MUP sample would be preferable.

Experience emerged as a clearer determinant of performance than specialty. Agreement with the consensus standard increased from fair among physicians with ≤1 year of practice to substantial–almost perfect among those with more than 10 years. The unexpectedly high performance of the 2–3-year group relative to the 4–9-year group is most plausibly explained by the small size and possible heterogeneity of the latter subgroup rather than by a genuine mid-career decline. Years of practice themselves remain only a surrogate for true experience; the volume of supervised studies required for competency recognition varies widely between countries (8), so that physicians with identical calendar service may differ markedly in cumulative interpretive exposure.

No statistically significant difference in agreement was observed between neurologists and clinical physiologists, although the comparison was underpowered. One systematic difference did appear: neurologists more frequently misclassified true myogenic recordings as neurogenic. This asymmetry may reflect the predominance of neurogenic pathology in neurological practice, which could bias borderline cases toward a neurogenic interpretation. Clinical physiologists, whose daily work more often incorporates quantitative motor-unit potential analysis against reference values, may be better calibrated to the normal–myogenic boundary. Nevertheless, the wide range of individual kappa values within both specialty groups indicates that personal competency is more decisive than formal specialty labels.

International surveys confirm that clinical neurophysiology is a primary specialty in only a minority of countries and is most commonly entered via neurology, physical and rehabilitation medicine or related fields, with substantial variation in training duration and content (8). Even within a single country, specialty titles convey limited information about actual EMG competence. A recent American Association of Neuromuscular and Electrodiagnostic Medicine (AANEM) survey reported that neurology residents received a mean of 8.4 weeks of EMG training compared with 21.3 weeks for residents in physical and rehabilitation medicine (9). Recognition of neurogenic changes appears to be taught effectively across pathways; the principal educational weakness lies in the identification of myopathic changes and of the boundary between normal and mildly abnormal findings. Addressing this gap requires greater emphasis on quantitative MUAP analysis (6), deliberate exposure to subtle myopathic cases, and structured feedback of the type used in milestone-based and simulation-supported curricula (9).

The present results support a shift toward competency-based rather than specialty-based credentialing, an approach already reflected in tiered training frameworks and in proposals for the global harmonisation of electrodiagnostic education (8, 9). A practical examination modelled on the design used here classification of a standardised set of recordings assessed against an expert consensus standard with a predefined minimum acceptable kappa threshold could serve as a feasible instrument for such competency-based certification.

## 5 Limitations

Several limitations should be noted. The sample of 30 recordings is relatively small for stable estimation of Cohen’s κ with three categories, resulting in wide confidence intervals, particularly in subgroup analyses. The uneven distribution of physicians across experience groups (*n* = 2, 10, 3 and 4) further limited statistical power; Table 4 is descriptive and does not support a formal learning-curve inference. A muscle-level analysis was not possible because archived datasets were labelled at patient level. The interference curve and force-graded recruitment were not shown; raters placed MUP markers and judged duration and amplitude only. This isolates reliability of MUP-based pattern assignment but reduces ecological validity relative to live EMG and is especially relevant to myopathy. Intra-rater reliability was not assessed. Three of the four experts who formed the CS had originally acquired a subset of the recordings. Although the CS session used anonymised files without clinical data and required consensus among four experts, residual familiarity with individual traces cannot be excluded and may have slightly inflated expert-level agreement. Six MUPs per muscle is fewer than the ~20 potentials recommended for formal quantitative MUAP analysis; this threshold was chosen because in severe neurogenic loss only a small number of motor units remain available for sampling. Sensitivity and specificity are descriptive pooled estimates from clustered data. The study was exploratory, and the non-significant difference between specialties must not be interpreted as equivalence given the low post-hoc power (≈ 45%). Demographic data and referring clinical diagnoses of the 30 source patients cannot be presented: the recordings were exported from the EMG software already anonymised, and linkage to the original clinical files is not possible. CS remains an electrophysiological reference standard rather than a nosological diagnosis.

## 6 Conclusion

In this exploratory study, needle EMG pattern classification demonstrated moderate to substantial agreement with an expert consensus standard. Neurogenic patterns were identified with high consistency, whereas differentiation between normal and myogenic patterns remained challenging. Greater clinical experience appeared to be associated with higher agreement, although this observation requires confirmation in larger cohorts. These findings support competency-based qualification and highlight the need for standardised training focused on the normal–myogenic boundary and on the limits of offline MUP-pattern reading.

## Data Availability

The original contributions presented in the study are included in the article/supplementary material, further inquiries can be directed to the corresponding author.
