# Supplementary material for: Inter-rater reliability of needle EMG pattern classification among physicians
Source: Front Neurol. 2026 Oct 15;17:1982862. doi: 10.3389/fneur.2026.1982862 (PMC13716625; doi:10.3389/fneur.2026.1982862)
Supplement: Supplementary file 1 [file Supplementary_file_1.docx]

# **Participant instructions: inter-rater agreement in needle electromyography**

*English translation of Supplementary File S1. Software screenshots are reproduced from the original file; on-screen labels remain in Russian.*

**Dear study participant,**

Thank you for agreeing to take part in the study assessing inter-rater agreement in needle electromyography (needle EMG).

1. **To begin, please do the following:**

Download the 30 studies containing motor unit potential (MUP) samples to a separate folder on your computer using this link: <https://disk.yandex.ru/d/OCpmUbyI2cAZZQ>

If needed, the same location also contains the installer for the latest version of the Neuro-MVP software (Нейро-МВП / Нейро-МПВ), which you can install on your computer.

*Fig. 1. Cloud folder with the 30 MUP studies and the Neuro-MVP installer.*

2. **Next, open the program** **NeuroMVP.Netomega.**

Go to Study Manager (Менеджер исследований), then select “Import study” (Импорт исследования).

*Fig. 2. Study Manager — Import study.*

3. **The study opens. It may contain one muscle or several muscles.**

*Fig. 3. An opened study. One or more muscles may be present.*

4. **To display markers on the waveform, click the MUP and then press Alt + left mouse button at the same time.**
5. **After reviewing all tabs (the different muscles) in the study, decide whether the sample corresponds to normal findings or to a particular pathological pattern, and enter this rating in the electronic questionnaire (after completing the identification section).**

The questionnaire is available at: <https://forms.gle/DGY8pFVVnnWNF4JJ6>

6. **Save the study.**

*Fig. 4. Save the study.*

7. **Close the study, making sure that the changes are saved.**
8. **Proceed to the next sample and repeat the steps above.**
9. **Export the marked studies to a separate folder.**

*Fig. 5. Export to a separate folder.*

*Fig. 6. Choose the destination folder for the exported studies.*

The folder containing the 30 samples in which you have placed the markers must be archived and returned — preferably as a link to cloud storage — to [e.seliverstova.md@gmail.com](mailto:e.seliverstova.md@gmail.com) **by 1 August 2024.**

**For any questions, please contact Ekaterina Gennadievna Seliverstova by email at** [e.seliverstova.md@gmail.com](mailto:e.seliverstova.md@gmail.com) **or on Telegram at @magneziya213.**
